# Medical and Physical Intelligence

**Published:** 1807-01

**Authors:** 


### MEDICAL AND PHYSICAL INTELLIGENCE.

J. Pierson, Esq. read the Croonian Lecture on Muscular Motion to the Royal Society this winter. It occupied the greater part of two evenings, in the course of which the lecturer entered into an elaborate detail concerning the heat and pulsations of animals in different latitudes, in order to ascertain their effect on their muscles. As an instance: in this climate the pulse of horses beat 36 times in a minute, that of cows 48, and that of men about 72; in Lapland, and other high northern latitudes, the human pulse does not beat more than from 45 to 50 times in a minute. Mr. P. has made numerous experiments on the muscles, in all which he found the muscular irritability completely destroyed by plunging them in water at the temperature of 96°; electricity, after such immersions, sometimes gave slight symptoms of excitability, but no human effort could ever again restore the muscular fibre to its proper tone and vigour. Cold produced similar effects on the muscular fibre, by instantly destroysng its irritability. Hence the necessity of great caution in applying warm water to the surface of bodies recently immersed in water in cases of suspended respiration, as heat may be equally as bad as cold, with regard to its effects on the muscular fibre, which by Mr. P. is considered in some degree the organ of life. Blood he regards as essential to life only as a stimulus to muscular irritability, and the abstraction of blood occasions death through the want of its stimulating powers to the muscles. The stomach he considers as the most important organ of the human frame, and its irritability is so excessive, that a blow on it will instantly destroy life, though the heart can support a wound some days.

Dr. Wollaston has invented a new portable blow-pipe for chemical experiments. It consists of three parts, so adapted to each other that they may be packed together, one within another. The interior tube is longer than the exterior, and the upper edge of the large end is turned outward, to diminish the effort of the lips requisite for retaining it in the mouth. The small extremity is placed obliquely, that the flame may be carried to a convenient distance from the eye.

The subject of the Bakerian Lecture, by Humphrey Davy, Esq. was on some Chemical Effects of Electricity. This ingenious chemist has proved, that even in distilled water there is combined both vegetable and animal matter, besides nitrogen gas and salt. Hence he has ascertained that electricity does not generate fixed alkali, but only evolves it.

*Theatre*

*Theatre, London Hospital.*

On the 20th of January, Mr. HEADINGTON and Mr. FRAMPTON will commence their Spring Course of Lectures on Anatomy, Physiology, and the Principles and Operations of Surgery.

Theory and Practice of Midwifery, Dr. DENNISON.

Surgery, Mr. HEADINGTON.

Anatomical Dissections and Demonstrations as usual.

*Theatre of Anatomy, Blenheim-Street, Great Marlborough-Street.*

The Spring Course of Lectures, on Anatomy, Physiology, and Surgery, will commence on Wednesday, January 21, at 2 o'clock in the afternoon, by Mr. BROOKES.

Spacious apartments, thoroughly ventilated, and replete with every convenience, will be open at eight o'clock in the morning, for the purposes of Dissecting and Injecting, where Mr. BROOKES attends to direct the students, and demonstrate the various parts as they appear on Dissection.

Pupils may be accommodated in the house.—Gentlemen established in practice, desirous of renewing their Anatomical Knowledge, may be accommodated with an apartment to Dissect in privately.

*Theatre of Anatomy, Great Windmill Street.*

Mr. WILSON's Spring Course of Lectures on Anatomy, Physiology, Pathology, and Surgery, will begin on Tuesday the 20th of January 1807, at two o'clock.

Dr. BADHAM's Spring Course of Lectures, on the Practice of Physic, Chemistry, and the Materia Medica, will be commenced on Monday morning, the 5th of January, at eight o'clock, at No. Clifford-street, New Bond-street.

Mr. BLAIR's Lectures on Anthropology: or, the Natural History of Man; illustrated by Anatomical Preparations, &c. (for the information of Scientific and Professional Gentlemen, Amateurs of Natural History, Students in the Liberal and Fine Arts, &c.) will re-commence on Tuesday evening, the 27th of January, at the Bloomsbury Dispensary, No. 62, Great Russel Street; to be continued every Tuesday and Friday evening.

Dr. CLARKE and Mr. CLARKE will begin their Lectures on Midwifery, and the Diseases of Women and Children, on Thursday, the 22d of January. The ectures are read at the house of Mr. Clarke, No. 10, Upper John-street, Golden-square, every morning, at a quarter past ten o'clock, for the convenience of students attending the Hospitals.

Dr. DENNISON and Dr. SQUIRE will, on Tuesday the 6th day of this month, begin a Course of Lectures on the Theory and Practice of Midwifery, and the Diseases of Women and Children.

Mr. GUNNING, Surgeon Extraordinary to his Royal Highness the Duke of Sussex, and Surgeon to St. George's Hospital, will commence his Spring Course of Lectures on the Principles and Practice tice of Surgery, at his house, No. 43, Conduit-street, Hanover-square, on Monday, the 19th of January next, at eight o'clock in the evening.

Mr. Moor, Surgeon Dentist to her Royal Highness the Duchess of York, will commence his Spring Course of Lectures, on the Structure and Diseases of the Teeth, on the 16th of February next, in which will be shewn the various operations requisite for them, and the Complete Practice of the Dentist explained.

Mr. Taunton will resume his Winter Course of Lectures and Demonstrations on Anatomy, Physiology, Pathology, and Surgery, on Saturday the 31st of January, 1807, at 8 o'clock in the evening, *precisely*, at 21, Greville-street. The Lectures will be continued, at the same hour, every Tuesday, Thursday and Saturday.

The first part of Dr. Clutterbuck's Inquiry into the Seat and Nature of Fever, is expected to make its appearance in the course of the month.

Dr. Herdman has in the press, his second Discourse on the interesting Subject of the Management of Infants, and the Treatment of their Diseases, written in a plain familiar style, to render it intelligible and useful to all Mothers, and those who have the Management of Infants.

Dr. Trotter's Work on Nervous Diseases, intitled, A View of the Nervous Temperament, is now in the Press, and will be published early in in 1807. From the nature of the inquiry, it has been found necessary to alter the title.

